# Sir George Newman Performs a Miracle

**Published:** 1918-08-17

**Authors:** 


### The Hospital

The Workers' Newspaper of Administrative Medicine and Institutional Life, Administration, National Insurance and Health.

No. 1680, Vol. LXIV. SATURDAY, AUGUST 17, 1918. PRICE TWOPENCE

### MEDICAL EDUCATION.

### SIR GEORGE NEWMAN'S MOMENTOUS MEMORANDUM.

The last place in which one would expect to find a broadminded, statesmanlike, thoughtful, unbureaucratic pronouncement is a publication, issued by a Department of the Government and presented to both Houses of Parliament by command of His Majesty. Such publications are as a rule mere dry-as-dust records of facts and accumulations of meaningless statistics, published only to gather dust upon upper shelves for a certain number of years, and then to meet their proper fate in the pulping vat. Sir George Newman has performed a miracle. He has addressed to the President of the Board of Education a memorandum of momentous importance. For the last ten years the Board of Education has been making grants in aid of medical education to medical schools in England and Wales, and in order to guide it in the method and direction of allocating these grants it has appointed a special Standing Committee to advise it. Of this Committee Sir George Newman, as Medical Assessor to the Universities Branch of the Board, is of course a member, and it has been his duty to visit the various medical schools to discover how the grants were expended. He has visited the principal medical schools in America, Canada, and Germany also, and thus has had almost unique opportunities "of considering and comparing, over a period of some years, the various methods adopted in the medical schools of this country and elsewhere for the training of the medical student," and he has embodied his conclusions in this momentous document.

The first impression its perusal leaves upon the mind is one of surprise and thankfulness that a Government office contains a man so wide-minded, so wholly free from the wooden rigidity, the hidebound inflexibility, the imperviousness to new ideas, that prevail in Government offices. Sir George Newman not only welcomes new ideas when they are proposed by others, but is himself an abounding, sparkling fountain of new ideas. We trust we shall do him no disservice with the Government Department that he serves when we apply to his memorandum, or notes, as he modestly calls them, the adjectives in the opening sentence of this article. In reading it we are refreshed as by a breeze of fresh moorland air in early morning. He reviews the whole system and scheme of medical education, from its earliest beginning in the secondary school, through all its stages and departments to the post-graduate studies of the established practitioner, and at every step he has some merit to recognise, some defect to point out, some suggestion of improvement to make, some noteworthy comment to add.

But it would give a very unworthy and imperfect notion of this most remarkable document to leave it to be understood that it deals only piecemeal with the several stages and departments of medical education. It does this, indeed, but it does much more. Sir George Newman takes a comprehensive and unified view of the whole subject, and insists throughout upon the necessity of co-ordinating and inter-relating the several subjects, preliminary, intermediate, and final, of the medical course, so that, instead of being taught and learnt as they are at present, in isolation from one another in watertight compartments, they shall be so brought into constant relation that each shall assist the other. When the student enters on his clinical studies he leaves behind him with relief and thankfulness his anatomy, physiology, and pharmacology, just as, when he entered upon these intermediate subjects, he made haste to discard and forget his knowledge of physics and chemistry and biology. The stifling, deadening hand of the examination system weighs heavily upon the whole curriculum. The student's chief, almost his only, aim is not the acquisition of knowledge, still less the acquirement of a method, and yet less again the training of his mind, but only the passing of the next examination; and when his final qualifying examination is passed he feels himself freed from an almost intolerable burden, and is, as a rule, so disgusted with the whole system that he has conceived a thorough distaste for further effort to learn. Sir George Newman would have him carry his anatomy, physiology, and pharmacology with him into the wards, and apply them as of course to every case he meets with. He would have him look upon his patient in the light of biology, as a living example illustrating biological truths, and affording instances, applications, and lessons in physics and chemistry. Only by the constant application of knowledge of the subsidiary and ancillary sciences is a case of disease to be thoroughly understood. Moreover, Sir George Newman lays his finger upon a cardinal defect in our system of medical education when he points out the utter neglect of the teaching of treatment. The whole effort of the clinical physician is to arrive at a diagnosis, which in too many instances merely means attaching a name to the patient's disease. When the diagnosis is arrived at, the task is complete. A perfunctory word or two may be said about treatment; a prescription may be written; but of close and accurate study of the result of the treatment prescribed, of following it up and noting the effects of the administration and withdrawal of drugs, nothing is done, nothing is said. Still less is there any instruction of treatment in the wider sense, of the regulation of diet, exercise, occupation, sleep, and the general regulation of life, which, taken together, are usually of far greater importance than the administration of drugs. In this matter especially our system of medical education is deficient.

But Sir George Newman is not concerned merely with the provision of practitioners skilled in the treatment of disease. The physician should be something more than this. "The medical man who has received an adequate education must have something more than learning. He must be also a skilled craftsman, and, knowing his way among men, he must be, in a general sense, a humanist." "The service of the public requires a well-informed and skilful doctor, but also an educated man, and before the professional training, and also as part of it, there must be a general equipment—adding not only to the pleasure and usefulness of life, but also to the standard and character of the medical training built upon it. . . The medical student is, and must be, utilitarian in purpose; but it is essential to his proper professional education that it should be deeply infused and inspired by the broadest and most liberal culture, from which he may derive mental discipline, scientific habit, and an intellectual mastery of the principles involved in his profession; what may be called a clear, calm, accurate vision and comprehension of all things bearing upon his calling, which shall give him not learning only, nor acquirements only, but the possession of the thought, the reason, and something of the philosophy of Medicine."

